# Study on correlation between TBM tailings gradation and granitic gneiss surrounding rock stability

**DOI:** 10.1038/s41598-023-47259-6

**Published:** 2023-11-18

**Authors:** Yuancheng Liu, Ronggui Deng, Bai Yang, Gang Cheng, Tuo Wang, Zhiqian Fu

**Affiliations:** 1 School of Civil Engineering, Southwest Jiaotong University, Chengdu, 610031 China; 2 Power China Chengdu Engineering Corporation Limited, Chengdu, 610072 China; 3 School of Architecture and Transportation Engineering, Guilin University of Electronic Technology, Guilin, 541004 Guangxi China

**Keywords:** Solid Earth sciences, Civil engineering

## Abstract

In the process of double-shield TBM excavation, it is difficult to directly observe and test the characteristics of the surrounding rock. In this paper, the screening test of the different type tailings in the wet state was carried out to obtain the gradation curve and curve evaluation index. Combining with the excavation parameters and the surrounding rock characteristics of the tailings, a comprehensive analysis was carried out to establish the evaluation system among with the tailings gradation characteristics, lithology characteristics and excavation parameters. The results showed that: Sparsely fissured rock: the tailings are extensional fractures, the tailings gradation curve is inverse “*S*” type and gentle, and the evaluation index value: (1.50 > lg(*Cu*) > 1.35), (1.90 > *C*_*c*_ > 1.10). Broken surrounding rock: the curve is “*L*” type and steep, the content of coarse particles is much more than that of fine particles and (1.10 > lg(*Cu*) > 1.00), (2.60 > *C*_*c*_ > 2.40). Fractured rock: the curve is “*Step*” type, the tailings particles lack the middle particle size, the minerals are mostly weathered, (2.15 > lg(*Cu* )> 1.95), (0.09 > *C*_*c*_ > 0.07). The research results have good applicability to the surrounding rock stability evaluation of the example tunnel, which verifies the feasibility of the method.

## Introduction

With the development of China’s western region, the construction of large and medium-sized transportation, water conservancy and hydropower and other infrastructure is increasing. However, due to the mountainous and plateau landforms in the western region, and the flatness requirements of traffic lines and headrace tunnels, the proportion of bridges and tunnels in the line is high. Among them, the total construction of long and large tunnels accounts for a large proportion. Long and large tunnels in this region generally have high ground stress, complex geological structure. The characteristics of rich groundwater and high ground temperature make the formation lithology and occurrence state of the tunnel crossing changeable, and adverse geological phenomena such as faults, high ground stress and cataclastic rock^1–4^ are common. From the perspective of economy and safety, TBM construction method is more used in long and large tunnels. However, for tunnels constructed with double shield TBM, due to the shielding of the cutter head and shield of the TBM, it is difficult to directly observe and test the rock mass morphology and stability in front of the tunnel face, which affects the operators’ accurate judgment of the characteristic grade of surrounding rock near the tunnel face. In order to effectively solve the problem that the double shield TBM can quickly and effectively evaluate the stability of surrounding rock in the construction process, scholars have carried out a series of research and practice.

Among them, Li et al.^5^ a multi factor comprehensive analysis method based on the principle of grey correlation degree was proposed, which mainly focused on the cyclic footage and the width of the excavation face, to evaluate the stability of the surrounding rock of the tunnel. Liu et al.^6^ proposed to carry out rebound and scale test in segment grouting holes, and established a discrimination method for the stability of surrounding rock under the closed working environment of double shield TBM. Deng et al.^7^ proposed a comprehensive method to identify the stability of surrounding rock of double shield TBM by using tailings lithology, rock block characteristics and characteristic size, rebound and measuring scale, which has good engineering application adaptability. Xu et al.^8^ proposes for the first time to use the tailings of TBM as the evaluation parameter to distinguish the characteristics of surrounding rock in the process of studying the geological logging of TBM, and gave the characteristics of tailings of four types of surrounding rock at different levels. Hou et al.^9^ comprehensively judged the surrounding rock category in Wanjiazhai Yellow River Diversion Project by using rock debris, window identification lithology, tunneling parameters and sound pea gravel backfill volume, and the site applicability was good. Yang et al.^10,11^ established a fuzzy theory for comprehensive evaluation of surrounding rock stability based on fuzzy theory, using evaluation parameters such as tunneling parameters, rock rebound and rock tailings analysis. Yan et al.^12^ established the relationship between TBM cutting tool wear and rock tailings grading distribution. Sun et al.^13^ and Wang et al.^14^ established the relationship between TBM operation parameters and tailings quality index based on regression analysis and fuzzy theory, and defined the service conditions under different geological conditions. Gong et al.^15^ and Li et al.^16^ combined TBM with intelligent recognition technology. Through the image statistics technology of rock tailings, the deep learning algorithm was used to segment and extract the image of tailings, and the real-time stability characteristics of surrounding rock were obtained. Zhang et al.^17^ established a comprehensive prediction method for geological conditions of roadheader based on big data analysis and taking the cutter head speed, torque, thrust and propulsion speed as indicators. However, the research on establishing the evaluation indicators of rock tailings grading curve, tunneling parameters and the correlation of surrounding rock integrity characteristics is not perfect.

On the basis of previous studies, based on the double shield TBM in the construction process, this paper analyzes and tests the rock tailings output by the belt conveyor in the wet state, such as the rapid particle sieve test, the judgment of the lithological characteristics of surrounding rock blocks and the statistics of the parameters of the TBM, and obtains the grading curve of the sample, the evaluation indicators of the grading curve, the lithological characteristics of surrounding rock and the parameters of the tunneling machine, The evaluation method of surrounding rock with different integrity of grading characteristics, lithological characteristics and tunneling parameters is preliminarily established.

## Project overview

### Overview of tunnel engineering

The site of *DXL* tunnel project is located in the plateau of Western China. The tunnel is a control project of multi-purpose highway, with the mileage pile number from K8 + 698 to K13 + 482. The total design length of the tunnel is 4784 m, the maximum burial depth of the tunnel body is 830 m, the elevation of the tunnel entrance is 3547.02 m, and the design longitudinal slope is 0.4%. The slope rises from the entrance to the exit, and the elevation of the tunnel exits is 3566.18 m. The inner diameter of the tunnel is designed to be 8.1 m, and the excavation diameter of the rough tunnel is designed to be 9.13 m. It is a single tunnel with two lanes. The double shield Tunnel Boring Machine construction method is selected for unidirectional excavation starting from the entrance according to the design.

**Figure 1:**
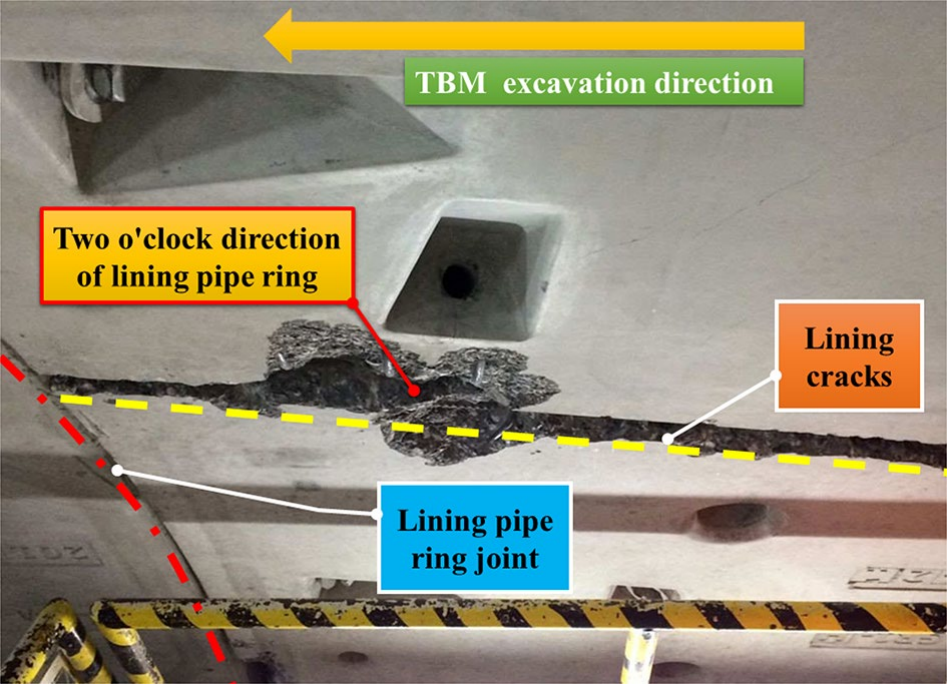
Longitudinal cracking of lining segment at two o’clock.

During the tunneling excavation of the tunnel, there were four times jammed accidents at the mileage from K10 + 076 to K10 + 253, which seriously interfered with the progress of the project. According to the preliminary observation and analysis after shutdown, serious internal extrusion cracking occurred in some sections of the lining, as shown in Fig. 1. The surrounding rock was broken. According to the previous geological survey and test, the TBM jammed accidents section has high geostress and tectonic action strongly developed. It can also be determined from the surrounding rock observed during shutdown that the rock mass fissures in this section are developed, and some bad diseases such as rock fragmentation occur in local sections. Therefore, in order to effectively avoid the frequent occurrence of TBM jammed accidents, a method to quickly obtain the integrity and stability of surrounding rock mass and working face during TBM construction is urgently needed, to provide decision support for engineers to adjust TBM tunneling parameters and attitudes in advance.

### Overview of tunnel engineering geological conditions and geological survey of the test section

The site is located in the southeast edge of the plateau in Western China, and the area is the intersection of the *Himalayas*, *Hengduan* Mountains and *Nyenchenthanglha* Mountains. Under the influence of regional strongly tectonic action, the tectonic phenomena such as rock mass crumpling, thrust fault and normal fault are extremely developed in the area. The overburden lithology is relatively simple, consisting of loose overburden composed of rock collapse fragments and moraines, and bedrock dominated by gneiss (Table 1). The mountain of the site is covered with snow all year round, and several snow melt lakes are scattered at both ends of the pass at the upper part of the tunnel. The surface and groundwater are sufficient, mainly bedrock fissure type and loose overburden pore type groundwater. Combined with the results of the preliminary geological investigation data of the tunnel and the supplementary detailed geological investigation data of the surrounding rock in the section after the four times TBM jammed accidents, the classification of surrounding rock by water conservancy and hydropower code^18^ as follows: the rock mass around the tunnel is of medium quality, and the rock mass unloading zone and fault zone at the entrance and exit slopes basically belong to class *V*; Some complete rock mass tunnel sections belong to class *II*; Most of the surrounding rock quality of the tunnel is class *III* and class *IV*. The maximum burial depth of the tunnel is 840 m, and the burial depth of the tunnel over 2 km in total of tunnel length reached 600 m.

**Table 1:** Lithology distribution characteristic table of tunnel site.

Rock mass lithology	Basic overview of lithology
Holocene alluvial, proluvial deposits	Sand, pebble gravel layer, covering layer thickness is less than 0.5 m, dense state
Holocene colluvial, clinosol deposits	Mainly boulder and gravelly soil, with covering layer thickness less than 20 m, large pores and poor compactness
Pleistocene glacial deposits	Boulder and gravelly soil, with good compactness, the burial depth less than 30 m
Granitic gneiss and mixed gneiss	It belongs to medium hard–hard rock, relatively developed gneisses obvious deformation. The average compressive strength is 72 MPa, and the rock is generally moderately hard to hard

This study mainly focuses on the three integrity types of sparsely fissured rock mass, fractured rock mass and fractured rock mass. Among them, the lithology of the above three types of rock mass is granite gneiss. The gneisses are relatively developed, the extension length of gneisses is large, the surface outcrop evolves into quasi planar structure, and the fractures are relatively developed. The laboratory mechanical test results show that: the average uniaxial saturated compressive strength of the fresh rock mass in the normal direction of gneissic schistosity is 82.5 MPa; the average uniaxial saturated compressive strength of the fresh rock mass in the parallel direction of gneissic schistosity is 72 MPa. The rock block is of medium to hard strength level.

## Tailings sieve test lithological characteristics evaluation and tunneling parameter statistics during tunneling excavation

### Test principle

In the excavation process of the double shield TBM advancing towards the tunnel face, the cutterhead of TBM is subjected to the thrust and torque of the oil cylinder, which makes the hob and scraper crush and scrape the rock mass respectively. Among them, the crushing effect of hob shows relatively stable and specific failure characteristics for rock masses with different lithology and weathering degree. At the same time, the response of the tunneling parameters of the TBM to the rock mass with different degrees of integrity will also show differences, which can be reflected in the parameters such as cutterhead penetration, torque, shoe pressure and shoe displacement. According to the characteristics of closed construction of double shield TBM, only the transported rock tailings of the surrounding rock mass information can be obtained directly during the construction process. Therefore, through the comprehensive analysis of the particle distribution characteristics and weathering state of rock tailings, and the change of tunneling parameters during excavation process, a comprehensive evaluation system of tailings grading characteristics, lithological characteristics and tunneling parameters can be established.

In this test, the rock tailings output by the belt conveyor is sampled immediately, and the rock tailings is screened under the wet state, and finally the grading curve and its characteristic evaluation parameter value are obtained. The particle size sampled in this test is greater than 0.05 mm, and the clay particles less than 0.05 mm are not statistically analyzed, and finally the grading curve of tailings greater than 0.05 mm is obtained. In this study, the non-uniformity coefficient ($C_{u}$) and curvature coefficient ($C_{{\text{c}}}$) are used to characterize the non-uniformity and continuity of rock tailings. Because the particle size of rock tailings fluctuates in a large range, in order to facilitate analysis and comparison, the common logarithm value of non-uniformity coefficient is defined, namely $\lg (C_{u} )$. In addition, in order to effectively define the overall thickness of rock tailings, $d_{50}$ is used as another index for evaluation. The three evaluation parameters are calculated as follows:

1 $$ {\text{Non-uniformity coefficient:}}\;C_{u} = \frac{{d_{60} }}{{d_{10} }}, $$

2 $$ {\text{Non-uniformity coefficient common logarithm value:}}\;\lg (C_{u} ) = \lg \left( {\frac{{d_{60} }}{{d_{10} }}} \right), $$

3 $$ {\text{Curvature coefficient:}}\;C_{{\text{c}}} = \frac{{d_{30}^{2} }}{{d_{60} \cdot d_{10} }}, $$

where $C_{u}$ the non-uniformity coefficient, $C_{{\text{c}}}$ the curvature coefficient, $\lg (C_{u} )$ the common logarithm value of non-uniformity coefficient, $d_{60}$ effective particle size of rock tailings, $d_{50}$ average particle size of rock tailings, $d_{30}$ intermediate particle size of rock tailings, $d_{10}$ boundary particle size of rock tailings.

### Evaluation on lithological characteristics of surrounding rock tailings of tunnel face

#### Characteristics of relatively complete surrounding rock tailings

The hob and scraper of TBM cutterhead conduct compression tension breaking and scraping and shearing breaking on the complete rock mass, making the rock tailings owning the characteristics of corresponding damage as follow:

1. The section of rock tailings is fresh and unweathered, and the minerals show their original color. Because the test object of this test is mainly granite gneiss, which is affected by the weakening of the integrity of the rock mass by the gneissic schistosity plane, and the rock block fracture surface is a relatively rough micro-step staggered fracture and tensile fracture section.
2. As the rock mass is relatively complete, the TBM cutter mainly carries out compression tension and scraping shear damage to the rock mass. The rock tailings fragments are mainly plate-like and schistose, and the fragments are mostly spindle shaped with both ends pinched out.

#### Characteristics of surrounding rock tailings with relatively developed fissures

For the surrounding rock mass with relatively developed joints and fissures, the cutterhead will not only perform compression tension breaking and scraping and shearing breaking on the complete surrounding rock mass, but also scrape off the rock block with the fissure surface cutting the enclosure. This type of surrounding rock tailings owns the following characteristics:

1. After the complete rock block cut and wrapped by the joint surface is destroyed, it also shows the same failure characteristics as the complete surrounding rock. In addition, due to the cutting of fissures, part of the cross-section of rock is exfoliated along the original fissures, so its color is of certain weathering characteristics, mainly dark-gray, mixed with the yellow of some weathered rock blocks.
2. Controlled by the joint plane, the rock block is cut by multiple sets of joint planes. Combined with the rock breaking effect of the cutterhead, the block retains the original joint cutting plane, which makes the rock fragments show blocky and polygonal shapes, so the block shows the characteristics of polygonal planes.

#### Characteristics of broken surrounding rock tailings

Broken surrounding rock such as fault zone and influence zone is seriously weathered, and the loose surrounding rock is directly planed by TBM cutter. The characteristics of rock tailings are as follows:

1. The rock tailings is mainly composed of fragments. The weathering trace on the surface of the gravel is obvious, and the surface of the rock block is grayish black.
2. The particle size distribution range of rock tailings is wide, the proportion of each particle group varies greatly, the content of fragments in the fine particle group is large, and the grading curve shows obvious step shape, indicating that some particle groups with intermediate particle size are missing.

### Experiment design and implementation

#### Sieve test of tailings in wet state

The round hole sieve is used in this experiment. The aperture combination is: 0.05 mm, 0.2 mm, 2 mm, 5 mm, 10 mm, 20 mm, 40 mm, 60 mm. Statistical analysis is not carried out for particle sizes less than 0.05 mm. Before the experiment, mark the basic information of the tunnel face, and randomly take out the rock tailings on the belt conveyor. Then the sieve test of rock tailings in wet state was carried out. The difference between this experiment and the sieve test in dry state is that water is injected at the top of the sieve lid to assist in screening. It may reduce the adhesion of particles caused by the surface tension of water film on the surface of wet rock tailings, and increase the fluidity of screened particles. After the screening is completed, the fragments in each tray are preliminarily dried on the surface, then are weighed, recorded and finally obtained the corresponding grading curve.

In this experiment, a total of 9 sieve tests were carried out on 9 samples in 3 groups of granite gneiss fragments of relatively complete surrounding rock, relatively developed fissures surrounding rock and broken surrounding rock. The relatively complete surrounding rock tailings samples number are: 1-1, 1-2, 1-3; the relatively developed fissures surrounding rock tailings samples number are: 2-1, 2-2, 2-3; Broken surrounding rock tailings samples number are: 3-1, 3-2, 3-3.

#### Setting tunneling parameters of the boring machine during testing

In order to obtain the grading characteristics of tailings corresponding to different integrity surrounding rocks during TBM excavation process.The excavation parameters of the excavation machine are limited to a certain range in the excavation testing section. So, the driving parameters are relatively fixed to ensure the single factor of the test results and the reliability of the test data. In this testing study, the excavation parameters were locked and controlled for the testing sections of sparsely fissured rock, relatively developed fissures rock and broken rock to obtain the tailings grading characteristic curves and parameters of the three types of surrounding rock. The excavation parameters of the DXL tunnel test section are shown in Table 2.

**Table 2:** Results of real-time tunneling excavation parameters of double shield TBM.

Parameter index	Penetration (mm/rot)	Torque (MN m)	Shoe pressure (bar)	Shoe displacement (mm)
Data range	5.60–7.80	2.60–2.85	156.00–158.00	287.00–289.00

## Experiment results and analysis

### Analysis of relatively complete surrounding rock tailings grading curve and surrounding rock characteristics

**Figure 2:**
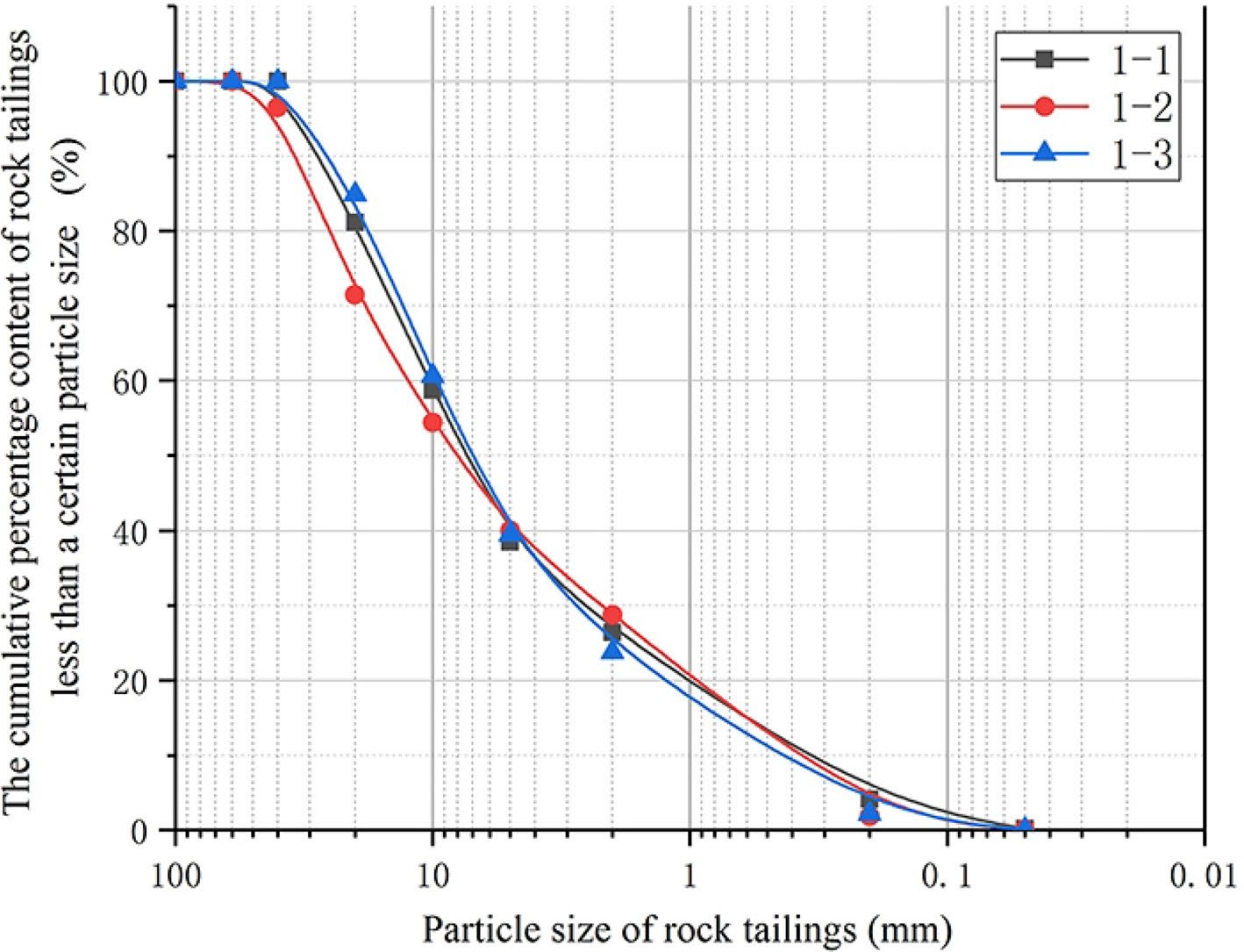
Grading curve of surrounding rock tailings of sparsely fissured rock.

There are three relatively complete surrounding rock test samples number: 1-1, 1-2, 1-3. The grading curves of the three samples are shown in Fig. 2. The grading curves of the three groups of relatively complete tailings samples own similar variation laws: the grading curves are relatively gentle as a whole, and the curves generally show the reverse “*S*” type variation characteristics. According to Table 3, the average particle size of rock tailings ($d_{50}$) ranges from 6.99 to 8.11 mm, with an average of 7.43 mm; The non-uniformity coefficient on the rock tailings curve is taken as the common logarithm value ($\lg (C_{u} )$) ranges from 1.34 to 1.49 mm. The particle size continuity of tailings is good, and the distribution of each particle group of rock tailings is relatively uniform; the curvature coefficient is 1.09–2.98, and the curve curvature is small.

**Table 3:** Test sample characteristic particle size and grading evaluation indicators summary table.

Type	Sample number	*d*_10_	*d*_30_	*d*_50_	*d*_60_	*C*_*u*_	lg* (Cu)*	*C*_*c*_
Sparsely fissured rock	1-1	0.34	2.56	7.20	10.58	29.95	1.49	2.98
	1-2	0.36	2.21	8.11	12.26	30.83	1.53	1.80
	1-3	0.43	2.80	6.99	9.47	33.70	1.34	1.09
Relatively developed fissures rock	2-1	2.27	12.63	21.50	26.45	11.63	1.07	2.65
	2-2	2.56	15.08	27.23	33.49	13.08	1.12	2.65
	2-3	3.76	18.01	29.76	36.60	9.74	0.99	2.36
Broken rock	3-1	0.06	0.18	4.62	6.21	103.63	2.02	0.09
	3-2	0.05	0.11	1.50	3.87	84.28	1.93	0.07
	3-3	0.07	0.22	6.89	9.46	142.21	2.15	0.08

**Figure 3:**
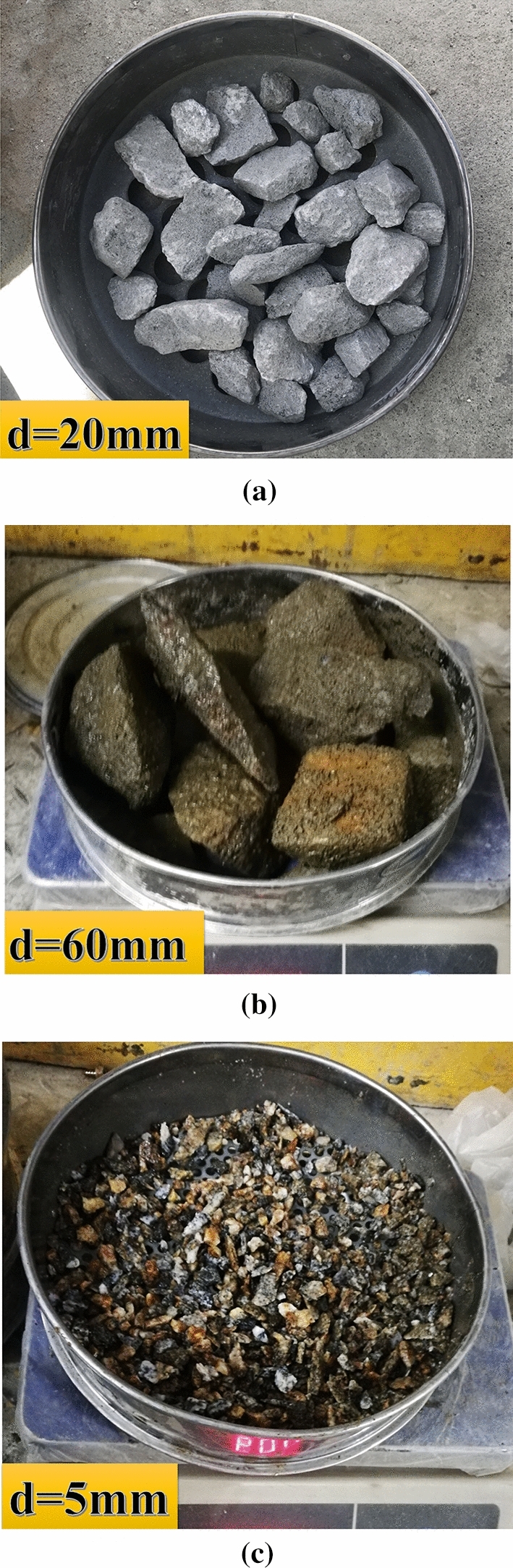
Lithological characteristics of three types of surrounding rock tailing: (**a**) characteristics of sparsely fissured rock tailings on 20 mm sieve mesh, (**b**) characteristics of relatively developed fissures rock tailings on 60 mm sieve mesh, (**c**) characteristics of broken rock tailings on 5 mm sieve mesh.

It can be seen from Fig. 3a that the characteristics of the residual rock tailings screened by the sieve mesh: the color of the rock tailings is gray, the rock surface is relatively fresh, showing the natural color of the original rock minerals without traces of weathering, and the block psephicity is poor. The rock blocks are angular, and most of them are long spindle shaped blocks. The cross-section of the block is relatively rough and distributed in an uneven micro step shape; at the same time, the backwater of excavation is gray. As the rock mass is relatively complete, with good integrity and undeveloped fractures, the damage effect of the cutterhead on the rock mass is mainly manifested as compressive tensile damage, and the cross-section of the rock block is mainly micro steps of tensile damage on the macro. According to the research Song et al.^19^, the damage of TBM disc hob to the rock mass of the tunnel face mainly includes extrusion, shearing and tensile damage, which will produce rock fragments, broken bodies and crushed bodies. The rock tailings formed by the three types of broken bodies correspond to different particle groups, making the particle grading of the complete rock tailings more continuous. The rock tailings of each particle group is distributed, and the lack of intermediate particle size is not obvious. Therefore, the grading curve of relatively complete surrounding rock tailings is characterized by a gentle reverse “*S-type*” without obvious steps.

### Analysis of rock tailings grading curve and surrounding rock characteristics of relatively developed fissures rock

**Figure 4:**
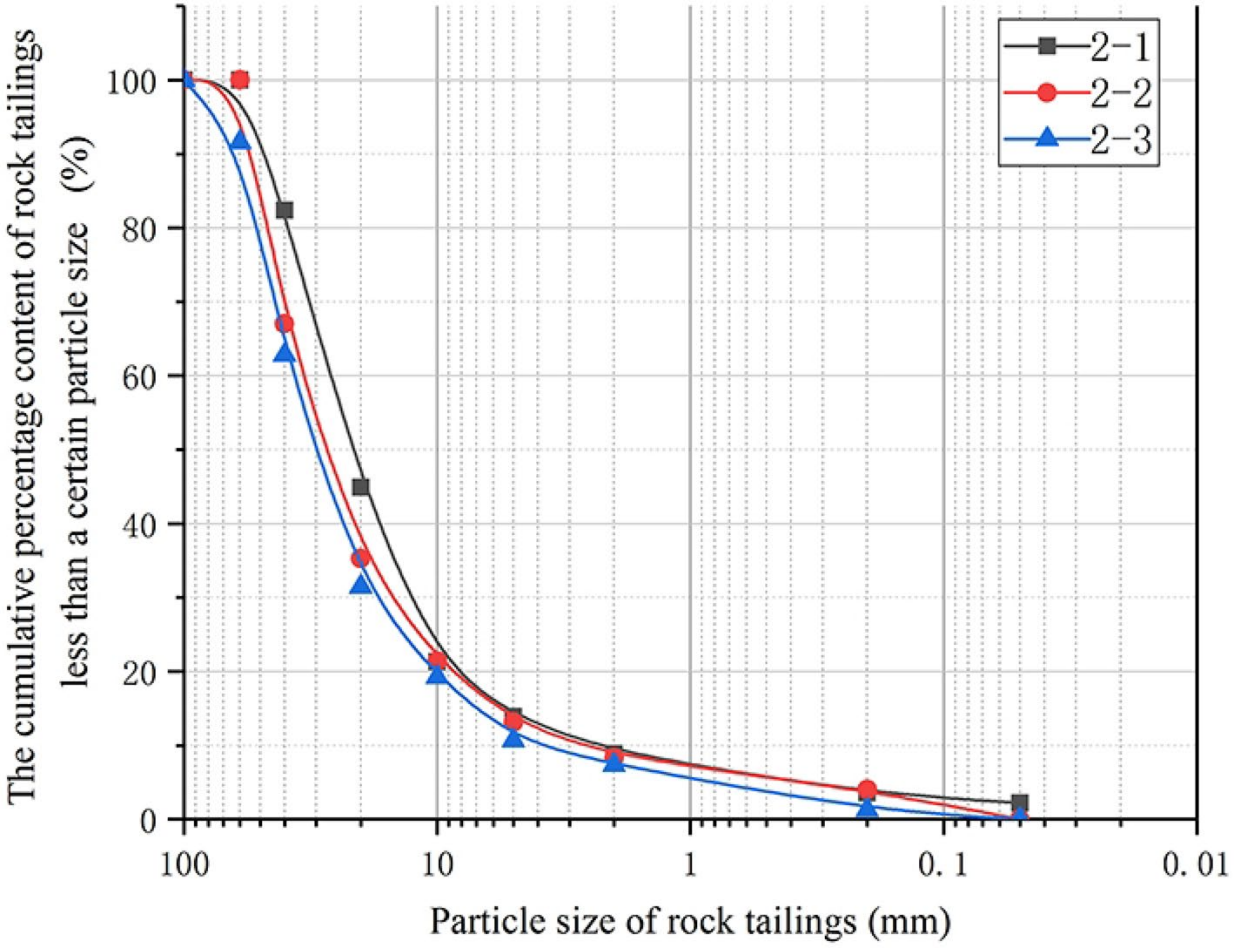
Grading curve of surrounding rock tailings of relatively developed fissures rock.

The sample numbers of rock tailings in surrounding rock with relatively developed fissures are: 2-1, 2-2, 2-3. According to the grading curves of the sieve test of the three samples in Fig. 4 and Table 3, the grading curve of the relatively developed fissures rock tailings generally has similar changes and generally presents the “*L-type*” change characteristics. The average particle size ($d_{50}$) corresponding to the cumulative weight of more than 50% of rock tailings in the three groups of samples is 26.16 mm, and the coarse-grained group of rock tailings particles accounts for a large proportion. The non-uniformity coefficient common logarithm value ($\lg (C_{u} )$) arranges from 0.99 to 1.12, with an average value of 1.06. It indicates that the distribution of tailings is concentrated in the range of coarse-grained group, and the proportion of fine-grained group is much lower than that of coarse-grained group. The curvature coefficient is in the range of 2.36–2.65, and the curvature is relatively large.

According to gradation curve in Fig. 3b, the rock tailings with a particle size over 60 mm has a high proportion. The rock tailings blocks are large. The blocks are plate-shaped and prismatic. There are weathering traces on some sections. The minerals on cross-section are quite different from the natural color of the parent rock, indicating that the cross-section minerals are weathered, which makes the minerals change qualitatively. In addition, the cross-section morphology of the blocks includes micro step shape and approximate plane shape. The backwater of excavation is gray. Among them, the minerals of most micro step sections are relatively fresh, and other sections are mostly weathered in yellow-black color. Compared with the tailings of sparsely fissured rock, some rock blocks have a certain degree of roundness and angular abrasion. In the surrounding rock mass with relatively developed fissures, the rock blocks cut by the primary fissures retain the overall structure of the complete rock mass and remain relatively stable under the unexcavated state. However, the groundwater and gas in the rock mass penetrate and corrode through the cracks of the rock mass, resulting in obvious weathering characteristics on the original structural plane of the fractured rock mass. Because this kind of rock mass still maintains a certain mosaic effect, the fracture erosion surface does not slide, so the rock block will only have a slight degree of erosion.

In addition, the cutterhead of the TBM cuts the surrounding rock, which destroys the initial stable structure of the fractured rock mass, and the mosaic effect of the whole rock block is damaged, making most of the rock mass slide and fall along the primary joint plane. The cutterhead can not cut this part of the rock mass enough, resulting that the particles of this type of rock tailings are generally large and relatively concentrated in the coarse-grained groups, while the content of the fine-grained group is relatively low.

### Analysis of rock tailings grading curve and surrounding rock characteristics of broken surrounding rock

**Figure 5:**
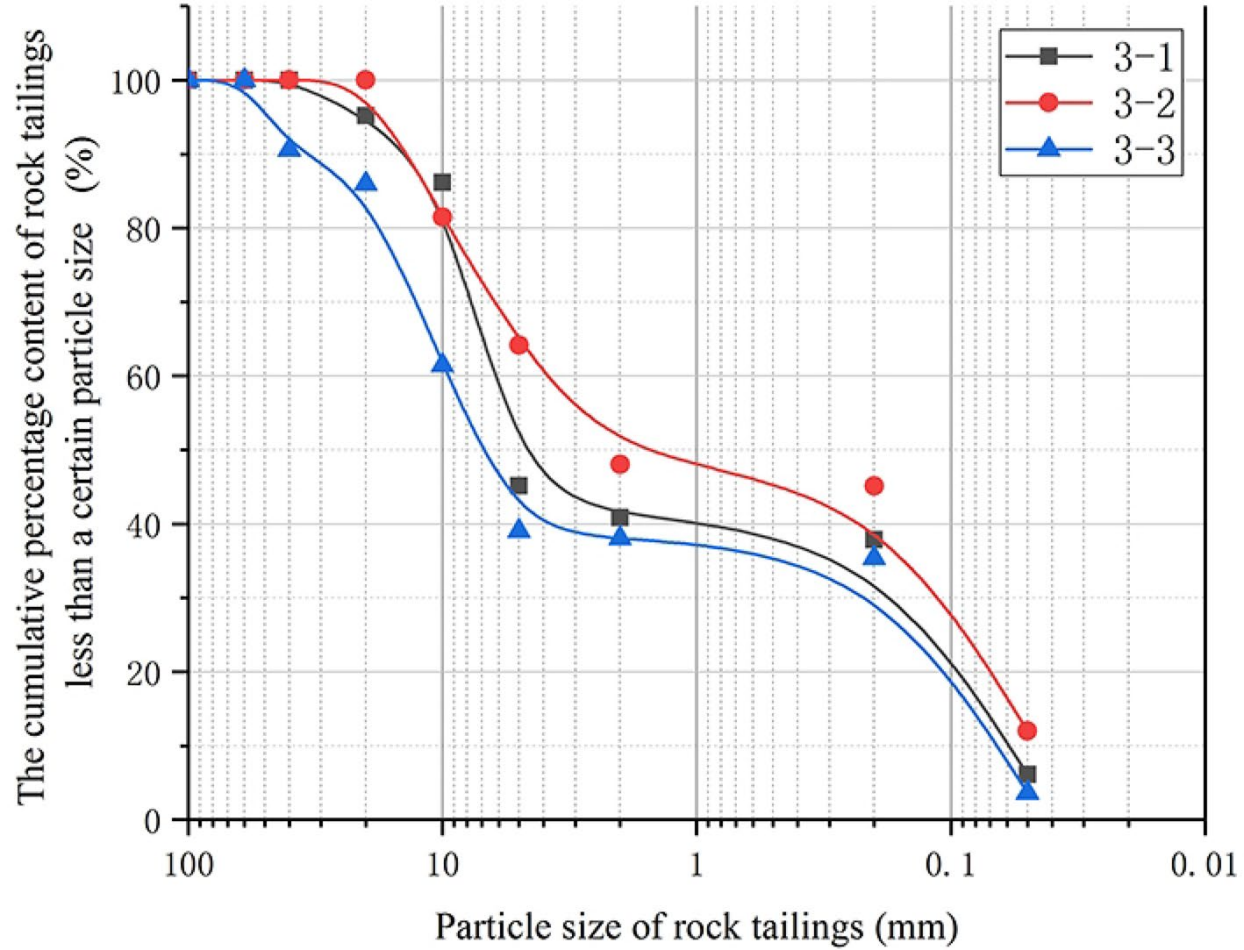
Grading curve of surrounding rock tailings of broken rock.

The samples numbers of broken surrounding rock tailings test are: 3-1, 3-2, 3-3. As Fig. 5 shown, for the test grading curves of the three samples, which are different from the characteristics of the grading curves of sparsely fissured rock’s and relatively developed fissures rock’s, and are in a “*Ladder-type*” shape as a whole. This kind of rock tailings presents an obvious curve platform, and the rock tailings has the characteristics of obvious lack of coarse-grain group, representing the characteristics of discontinuous grading. In addition, Table 3, shows that the average particle size ($d_{50}$) of this kind of rock tailings is in the range of 1.50–6.03 mm, with an average of 4.05 mm. Compared with the first two types of rock tailings, the content of fine-grained group is higher. While the non-uniformity coefficient common logarithm value ($\lg (C_{u} )$) is in the range of 1.93–2.15, with an average value of 2.03. It indicates that there is a large difference between the coarse-particle and fine-particle groups. The curvature coefficient is in the range of 0.07–0.09. According to Fig. 3c, the tailings particles of broken rock present obvious yellow color, and the weathering of the particles is serious. The rock tailings is generally in the shape of small fragments, and the particles have obvious weathering traces and roundness. The backwater water is mixed by yellow and black impurities.

Before excavation, the broken rock mass is in a relatively loose accumulation weathering state. The particles are weakly cemented by chemical weathering products, and the particles are relatively fine. The long-term erosion of groundwater and pore water leads to the particles become disintegrated and fine. The physical and chemical weathering effects are obvious. The particles are relatively fine, mainly granular and spherical, and have a certain degree of roundness. The particle surface has the unique yellowing characteristics of continuous weathering. The damage effect of the cutterhead on this kind of rock mass is mainly planing, and only a few large rock blocks have the characteristics of compression-shear damage. Therefore, the tailings of this kind of rock mass are mainly weathered particles, and are mainly fine particles mixing with a large number of weathered solutes. In addition, there are also some large rock masses in this type of rock mass. Therefore, the coarse-grained group formed by incomplete shear failure of cutter head will occupy a certain number, and the intermediate-grain group has not been completely formed. As a result, the coarse and fine grain groups respectively occupy a certain proportion in the grading curve, but there will be the absence of intermediate-particle size, that is, forming a platform of the grading curve.

### Analysis of grading evaluation indicators of three types of tailings grading curve

**Figure 6:**
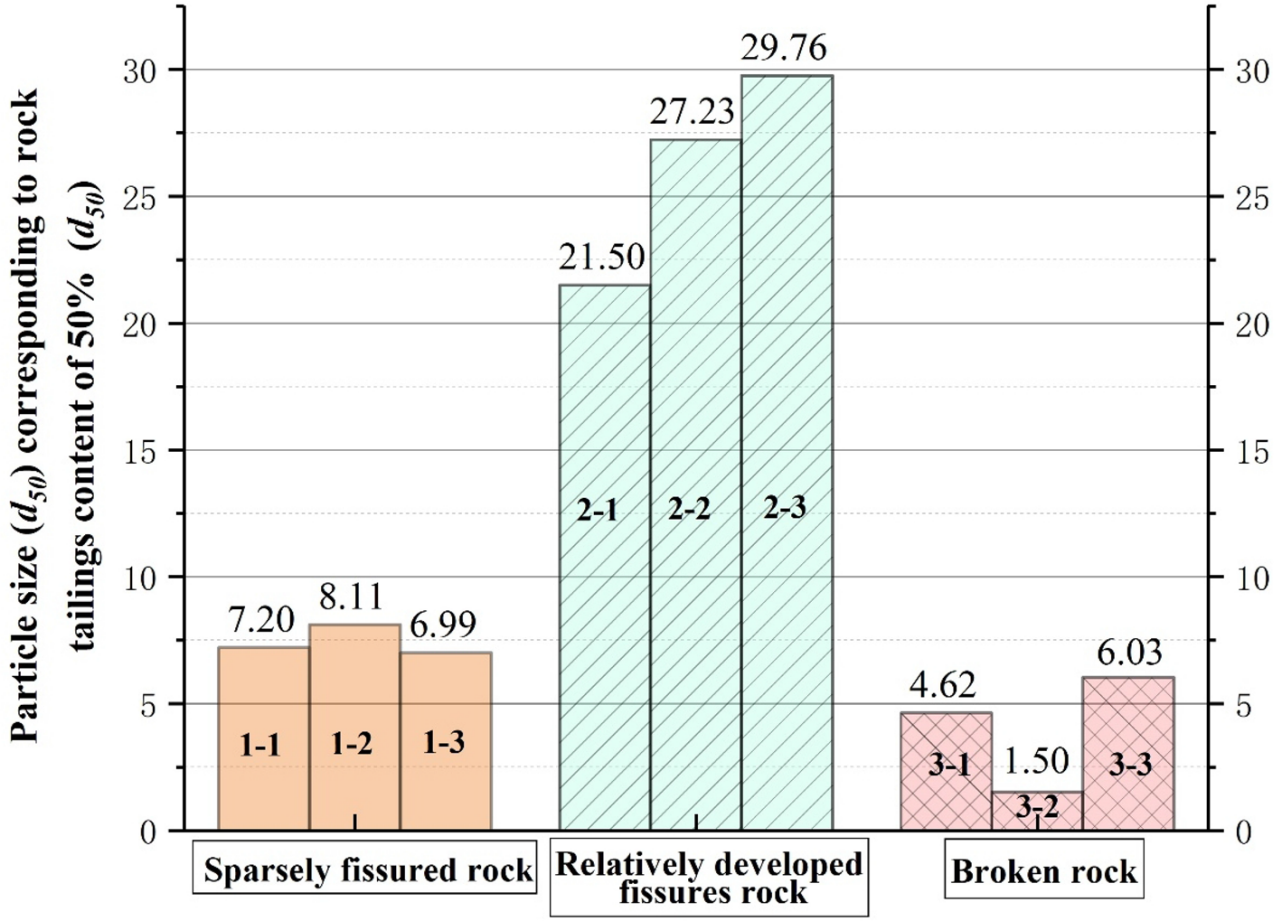
Average particle size (*d*_50_) distribution of three kinds of testing rock tailings samples.

**Figure 7:**
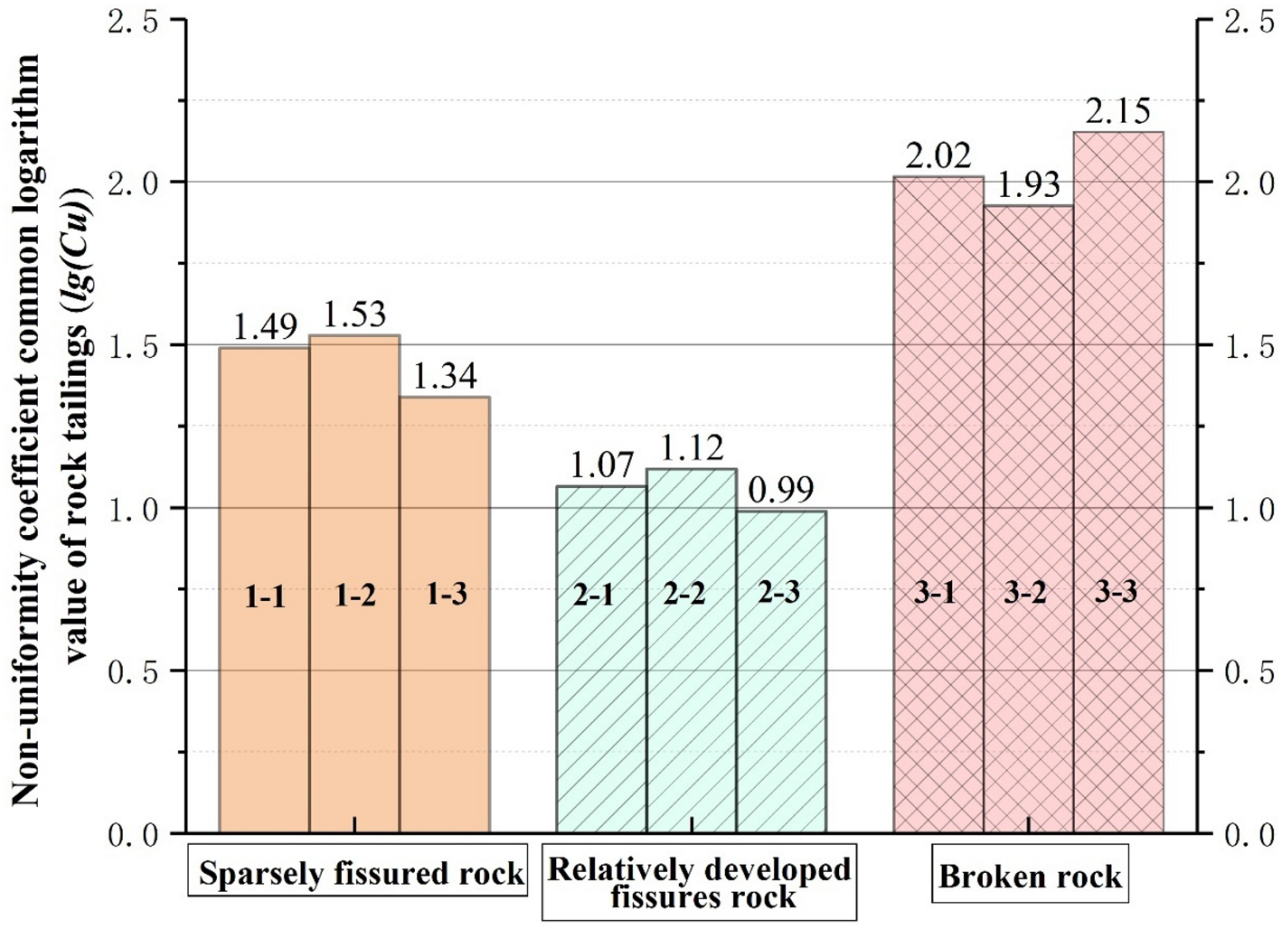
Distribution of the non-uniformity coefficient common logarithm value (lg(*C*_u_)) of three kinds of testing rock tailings samples.

**Figure 8:**
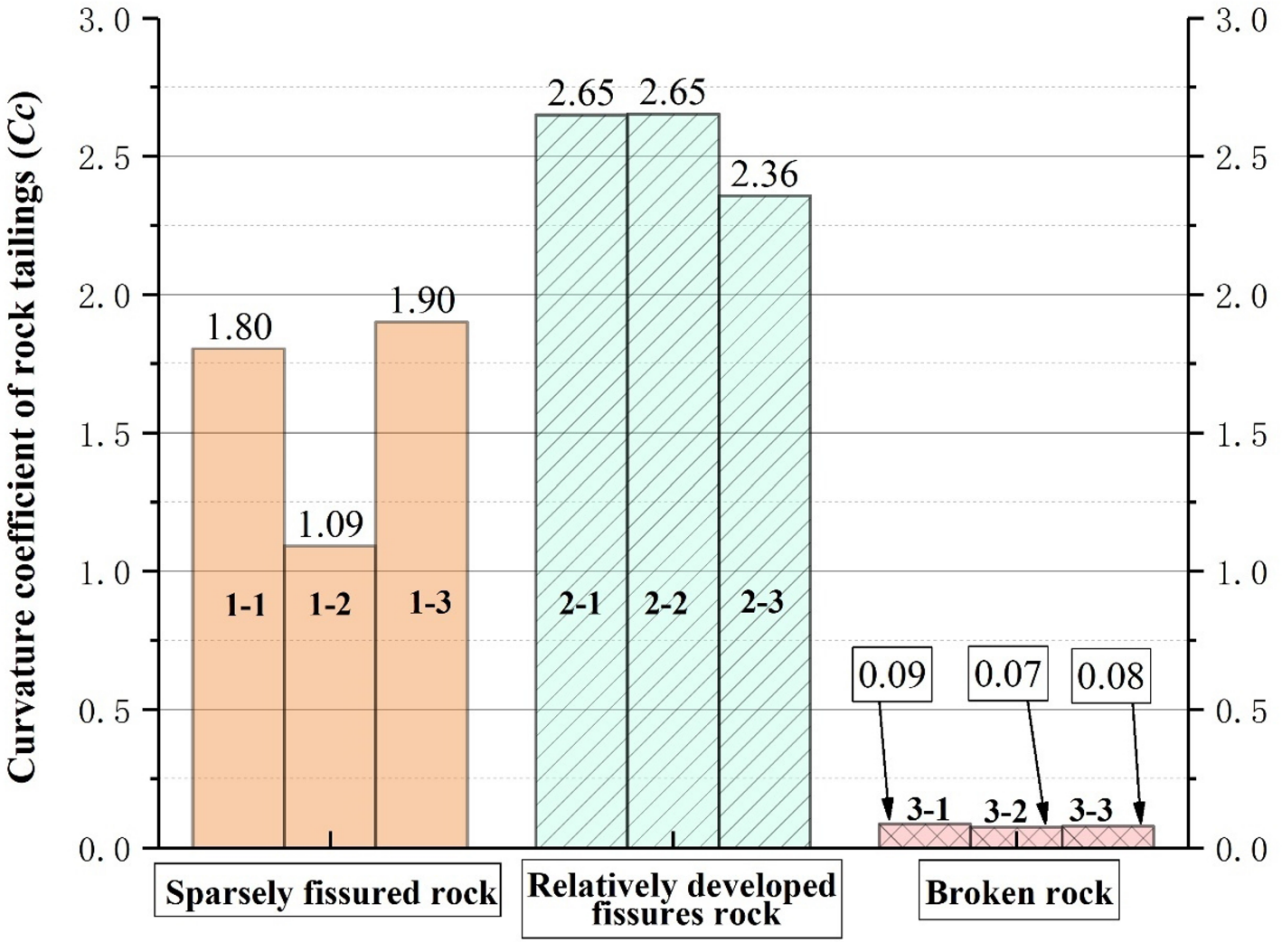
Distribution of curvature coefficient (*C*_*C*_) of three kinds of testing rock tailings samples.

According to the above research, the grading curves of the three types of rock tailings tests have their own specific curve characteristics. In order to further study the particle characteristics of the three types of rock tailings, this study selects three evaluation indicators: the average particle size ($d_{50}$), the non-uniformity coefficient common logarithm value ($\lg (C_{u}$) and the curvature coefficient (*C*_*c*_). The statistical results are shown in Table 3, Figs. 6, 7 and 8. First of all, according to Figs. 6, 7 and 8, in the same evaluation indicators ($d_{50}$, $\lg (C_{u}$, *C*_*c*_). The rock tailings of different lithology has its own value range without intersection, reflecting the specific value range of different types of rock tailings. Therefore, the evaluation indicators can be further analyzed to establish the corresponding relationship between the evaluation indicators of the tailings grading curve of different integrity of surrounding rock.

According to Fig. 6, the average particle size ($d_{50}$) of three types of rock tailings is 6.99–8.11 mm, 21.50–29.76 mm and 1.50–6.13 mm for relatively complete surrounding rock, relatively developed fissures rock and broken rock respectively. Mainly due to the influence of surrounding rock fissures and weathering degree, different types of rock mass response different particle sizes during TBM cutterhead excavation. For the granite gneiss in this study, the average particle size of tailings ($d_{50}$) and its reference value during TBM excavation are as follows: fractured rock mass (21.50–29.76 mm) > sparsely fissured rock mass (6.99–8.11 mm) > broken rock mass (1.50–6.13 mm).

According to Fig. 7, the non-uniformity coefficient common logarithm value ($\lg (C_{u}$) of three types of rock tailings are also different, and the test values are: broken rock mass (1.93–2.15) > sparsely fissured rock mass (1.34–1.54) > fractured rock mass (0.99–1.12). Similarly, for the three types of rock tailings, the curvature coefficient (*C*_*c*_) are: fractured rock mass (2.36–2.65) > sparsely fissured rock mass (1.09–1.90) > broken rock mass (0.07–0.09).

To sum up, the average particle size (*d*_50_) of tailings varies according to different geological conditions, which cannot be quantitatively defined. However, the relative particle size can be determined based on the statistical analysis of multiple groups of test results in the tunnel site. The non-uniformity coefficient common logarithm value (lg(*C*_u_)) and the curvature coefficient (*C*_*c*_) can be usually used as a reference value. It is corrected according to the test data and retains a certain degree of reliability. The reference values can be referenced as follows:

1. Sparsely fissured rock tailings:
  (1.50 > lg(*C*_*u*_) > 1.35), (1.90 > *C*_*c*_ > 1.0).
2. Relatively developed fissures rock tailings:
  (1.10 > lg(*C*_*u*_)>1.00), (2.60 > *C*_*c*_ > 2.40).
3. Broken rock tailings:
  (2.15 > lg(*C*_*u*_) > 1.95), (0.09 > *C*_*c*_ >0.07).

### Analysis of the corresponding driving parameters to the three types of rock tailings

According to Fig. 3a, as the integrity of surrounding rock changes from integrity to fragmentation, the penetration of cutterhead increases obviously; the torque of the cutterhead is greatly decreased. The integrity of the complete surrounding rock is good, and the cutterhead can only move forward after breaking the rock and the penetration degree is low; Due to the influence of the cutting surface of fractured rock mass, the rock mass is easier to be scraped off than that of complete rock, so the penetration degree increases slightly. Compared with the other two types of rock mass, broken rock mass has loose surrounding rock mass, maximum penetration degree, but minimum torque. According to Fig. 3b, from sparsely fissured rock, fractured rock to broken rock, the shoe pressure and displacement also show a certain reverse change law. The more complete the surrounding rock, the greater the shoe pressure and the smaller the displacement. This law has a good identification characteristic that indirectly reflects the integrity of surrounding rock.

### Comprehensive evaluation method for real-time stability of surrounding rock in TBM excavation process

According to the research results in the article, under the conditions of similar TBM operation parameters, the tailings grading curves of different stability types of surrounding rock present different shapes, the variation range of the grading parameters is also in the range of values that do not coincide. From this, combined with the lithological characteristics of the tailings, an indirect and comprehensive method for determining the stability of surrounding rock can be obtained. This study is based on the TBM tunneling project of DXL tunnel. According to the research results of this paper, under the conditions of similar tunneling parameters, the criteria for determining the stability of different surrounding rocks in real time can be obtained:

1. *Sparsely fissured surrounding rock* The tailings grading curve is gentle and presents an “S” shape. The range of grading curve parameters is: (1.50 > lg (Cu) > 1.35), (1.90 > Cc > 1.0). Lithological characteristics of tailings: the minerals of rock are fresh, low weathering degree, rough rock surface, and the tailings are angular.
2. *Relatively developed fissures surrounding rock* The tailings grading curve is in a “stepped” shape, and the range of grading curve parameters is: (1.10 > lg (Cu) > 1.00), (2.60 > Cc > 2.40). Lithological characteristics of tailings: There are partial weathering characteristics in rocks minerals, and the tailings appear partly yellow on the rock surface. The size of the tailings rock blocks is polarized, lacking of intermediate particle size of tailings. The structural planes of tailings are obvious.
3. *Broken surrounding rock* The tailings grading curve is steep and presents an “L” shape. The range of grading curve parameters is: (2.15 > lg (Cu) > 1.95), (0.09 > Cc > 0.07). Lithological characteristics of tailings: The weathering degree of rocks minerals is high, and the tailings are also yellow and black in color, with unclear edges and different shapes.

## Conclusion

In this paper, based on the double shield TBM excavation in granite gneiss section, analyses the particle screening experiment statistics, lithology identification on the belt conveyor and tunneling parameter characteristics. Three kinds of grading curves with different degree of integrity and grading curve evaluation indicators—rock characteristics of surrounding rock-tunneling parameters variation rule are obtained, which certain reference significance for identifying the stability of granitic gneiss in time and effectively. The grading curve characteristics and lithological characteristics of three types of rock mass can be summarized as follows:

1. The grading curve of rock tailings of relatively complete surrounding rock mass generally presents an inverse “*S*” shape, the curve is gentle and the tailings particle size distribution is relatively uniform. The backwater during tunneling is mostly gray; the lithology of the rock tailings is the natural color of the parent rock, with few traces of weathering; the rock tailings particles are angular, and the large particles are mostly spindle shape, with poor roundness. The non-uniformity coefficient common logarithm value is: (1.54 > $\lg (C_{u} )$ > 1.34), and the Curvature coefficient is: (1.90 > $C_{c}$ > 1.09). The cutterhead penetration and shoe displacement take the lower value; the torque and shoe pressure take the higher value.
2. The grading curve of the rock mass with relatively developed fissures generally presents an obvious “*L*” shape. The backwater during tunneling is gray. The average particle size ($d_{50}$) is 26.16 mm, and the proportion of coarse-grained group is much larger than that of fine-grained group. The rock tailings block size is large, mostly plate-shaped and prismatic, and some sections have weathering traces, the overall rock tailings presents yellow-black color, and some rock blocks have certain roundness and angular abrasion. The non-uniformity coefficient common logarithm value is: (1.12 > $\lg (C_{u} )$ > 0.99), and the Curvature coefficient is: (2.65 > $C_{c}$ > 2.36).
3. For the Broken rock tailings, the grading curve appears a platform that interrupts the continuity of grading, and the curve is similar to the “*Step*” shape. The coarse and fine particles of rock tailings can be distinguished obviously, and part of the intermediate grain group is missing. The mineral weathering is serious, and obvious weathering products appear. The surface of the block is yellowing. The backwater during tunneling is yellow and mixed with black weathered solutes. The surface of rock particles has obvious rounded characteristics. The non-uniformity coefficient common logarithm value is: (2.15 > $\lg (C_{u} )$ > 1.93), and the Curvature coefficient is: (0.09 > $C_{c}$ > 0.07). The cutterhead penetration and shoe displacement take higher values; the torque and shoe pressure take the lower value.
4. In this study, the tailings are quickly wet screened to quickly identify the lithological characteristics of rock tailings. It can provide certain evaluation parameters for timely and effective evaluation of the lithological stability characteristics of the tailings at the current tunneling position. Combined with the results of this study, it can be adopted: type of grading curve, average particle size ($d_{50}$). The non-uniformity coefficient common logarithm value ($\lg (C_{u} )$), the Curvature coefficient ($C_{c}$) and lithology characteristics of rock tailings. Based on the above evaluation indicators, the stability of surrounding rock can be evaluated effectively.
5. This study focuses on the testing of tailings with a particle size greater than 0.05 mm in a wet state, but there is a lack of testing for tailings with a particle size less than 0.05 mm. In subsequent research, a rapid method for obtaining testing parameters can be developed for particle size less than 0.05 mm of the tailings, so that the grading curve and parameters of the tailings can be completed.

## Data Availability

The data used to support the findings of this study are available from the corresponding author upon request.
